# Unique transcriptional signatures correlate with behavioral and psychological symptom domains in Alzheimer’s disease

**DOI:** 10.1038/s41398-024-02878-z

**Published:** 2024-04-04

**Authors:** Daniel W. Fisher, Jeffrey T. Dunn, Rachel Keszycki, Guadalupe Rodriguez, David A. Bennett, Robert S. Wilson, Hongxin Dong

**Affiliations:** 1 Department of Psychiatry and Behavioral Sciences, Northwestern University Feinberg School of Medicine, Chicago, IL 60611 USA; 2 Department of Psychiatry and Behavioral Sciences, University of Washington School of Medicine, Seattle, WA 98195 USA; 3 Mesulam Center for Cognitive Neurology and Alzheimer’s Disease, Northwestern University Feinberg School of Medicine, Chicago, IL 60611 USA; 4 Rush Alzheimer’s Disease Center, Rush University Medical Center, Rush University Medical Center, Chicago, IL 60611 USA

**Keywords:** Molecular neuroscience, Learning and memory

## Abstract

Despite the significant burden, cost, and worse prognosis of Alzheimer’s disease (AD) with behavioral and psychological symptoms of dementia (BPSD), little is known about the molecular causes of these symptoms. Using antemortem assessments of BPSD in AD, we demonstrate that individual BPSD can be grouped into 4 domain factors in our cohort: affective, apathy, agitation, and psychosis. Then, we performed a transcriptome-wide analysis for each domain utilizing bulk RNA-seq of post-mortem anterior cingulate cortex (ACC) tissues. Though all 4 domains are associated with a predominantly downregulated pattern of hundreds of differentially expressed genes (DEGs), most DEGs are unique to each domain, with only 22 DEGs being common to all BPSD domains, including *TIMP1*. Weighted gene co-expression network analysis (WGCNA) yielded multiple transcriptional modules that were shared between BPSD domains or unique to each domain, and NetDecoder was used to analyze context-dependent information flow through the biological network. For the agitation domain, we found that all DEGs and a highly associated transcriptional module were functionally enriched for ECM-related genes including *TIMP1, TAGLN*, and *FLNA*. Another unique transcriptional module also associated with the agitation domain was enriched with genes involved in post-synaptic signaling, including *DRD1, PDE1B, CAMK4*, and *GABRA4*. By comparing context-dependent changes in DEGs between cases and control networks, *ESR1* and *PARK2* were implicated as two high-impact genes associated with agitation that mediated significant information flow through the biological network. Overall, our work establishes unique targets for future study of the biological mechanisms of BPSD and resultant drug development.

## Introduction

Although dementia is largely defined by memory and cognitive decline which results in the loss of the ability to function independently, behavioral and psychological symptoms of dementia (BPSD) play a large role in a patient’s overall functional level [1]. Significant BPSD are more the rule than the exception, as >90% of people with dementia develop BPSD during the disease course [2, 3]. BPSD encompass a wide array of symptoms and include aggression, agitation, hyperactivity, compulsions, disinhibition, anxiety, depression and dysphoria, euphoria, delusions, and hallucinations. In addition, there is evidence that mild behavioral impairment is the first sign of dementia in some people, analogous to the more widely recognized mild cognitive impairment (MCI) [4]. In agreement with this, neuropsychiatric symptoms can precede a dementia diagnosis [5], with some estimates suggesting that over half demonstrate neuropsychiatric symptoms before a diagnosis of a cognitive disorder, including MCI [6]. Neuropsychiatric symptoms are also associated with faster cognitive decline in clinically normal older individuals [7, 8] and those with MCI [9], quicker development of dementia in people with MCI [10, 11], and faster dementia progression [12]. In addition, BPSD result in poorer quality of life for those with dementia [13, 14] as well as significant caregiver distress, often greater than with cognitive deficits [15, 16]. Emergent and difficult to treat, BPSD are often the cause of hospitalization and institutionalization for persons with dementia [17, 18]. Behavioral interventions remain the first line for treating any BPSD, and there is only one FDA-approved medication that is indicated to treat any BPSD [19, 20].

BPSD are a heterogeneous group of symptoms, and while each symptom has its own frequency of presentation, studies have suggested that certain symptoms co-occur at greater rates than others [21, 22]. In particular, a systematic review of 62 studies utilizing unbiased clustering of BPSD found that affective symptoms (dysphoria, anxiety), apathy, hyperactivity-impulsivity-disinhibition-agitation-aggression, and psychosis (delusions, hallucinations) all tend to form independent clusters [22]. This could implicate similar molecular mechanisms underlying each cluster.

Despite the ubiquity and burden of BPSD clinically, very few molecular studies exist to elucidate the molecular mechanisms underlying BPSD [23, 24], with the exception of AD with psychosis [25, 26]. To begin to identify the molecular mechanisms of BPSD in this study, we first verified the occurrence of BPSD domains in a sample of older adults based on the use of a structured clinical interview administered within two years of death. Then, we confirmed the clustering of BPSD into four predominant domains and performed bulk RNA-seq analysis on the post-mortem anterior cingulate cortex (ACC) from a subset of those with AD and varying burdens of BPSD. Further, we performed weighted gene co-expression network analysis (WGCNA) to identify transcriptional clusters associated with each BPSD domain. Finally, we used an algorithm to assess context-dependent information flow differences between the transcriptional networks of cases and controls to determine likely molecular drivers behind each BPSD domain. Our data set aims to better understand these four BPSD domains on the transcriptional level and yield promising targets for future mechanistic studies and novel therapeutics.

## Methods

### Subjects

Community-dwelling older adults who later developed dementia and their informants were recruited through the Rush Alzheimer’s Disease Center (RADC) memory clinic. As a part of their research visits, trained research assistants conducted standardized clinical interviews via telephone with informants, including an assessment of the frequency and severity of numerous BPSD. Questions pertaining to BPSD were developed by two neuropsychologists from clinical descriptions in the literature, observations of patient behaviors, and caregiver interviews, as described previously [27–30].

This study was approved by an Institutional Review Board of Rush University Medical Center (RUMC). For all subjects, consent for brain autopsy was obtained after death from next of kin and a witness by RUMC staff. Inclusion criteria for subjects in the present study were based on the NINCDS-ADRDA clinical criteria for “probable AD” with diagnosticians blinded to post-mortem findings [31]; all subjects had a history of cognitive decline, impairment in memory and at least one other cognitive domain, and no other conditions judged to be probably contributing to cognitive impairment (e.g., stroke, Parkinson’s disease). Following autopsy, all subjects underwent neuropathological evaluation by a neuropathologist who was blinded to clinical diagnosis. Subjects received a modified (i.e., dichotomized) NIA-Reagan score based on Braak staging of neurofibrillary tangles and CERAD scoring of neuritic plaques [32, 33]. In total, we identified 192 subjects with probable AD dementia due to primary AD neuropathologic change.

### BPSD domains

Longitudinal, structured clinical interviews of BPSD were completed between January 1992 and September 2005 during research visits through the Rush Alzheimer’s Disease Research Center. Trained research assistants conducted standardized clinical interviews via telephone with informants, who were defined as the person with the greatest amount of daily contact with each subject. As part of these interviews, informants were asked to rate the presence/absence, frequency, and severity of BPSD over the past month. Questions about affective symptoms (i.e., depression and anxiety) were based on but did not fully reproduce the Hamilton Depression Rating Scale [34]. This widely used measure has high interrater reliability (0.82–0.98) and test-retest reliability (0.81–0.98), adequate internal reliability for most items, and adequate convergent and discriminant validity [35]. Questions regarding delusions, hallucinations, agitation/aggression, and apathy were based on the Rush Patient Behavior Checklist, which was developed by two neuropsychologists on 146 community-dwelling patients with dementia who met NINCDS-ADRDA clinical criteria for “probable AD” [29]. The creation of questions about delusions and hallucinations were based on DSM-III-R criteria [36]. Symptoms of agitation/aggression and apathy were based on clinical descriptions in the literature, observations of patient behaviors, and caregiver interviews. Subscales of the Rush Patient Behavior Checklist show high interrater reliability (0.88–0.99) and adequate internal consistency (0.63–0.86) [27]. Studies have also shown relationships between The Hamilton Depression Rating Scale and the Rush Patient Behavior Checklist to diverse patient outcomes in patients with probable AD, suggesting strong external validity. Specifically, informant ratings on these measures are related to rates of institutionalization [37], level of cognitive decline [29, 30], degree of functional impairment [27], and mortality [30] in AD patients.

To determine the grouping of BPSD into domains, we performed a principal component analysis with oblimin rotation on responses to BPSD questions (Supplementary Table S1) from 192 patients with dementia due to AD. These BPSD questions were Likert scored (see “sample selection” for more details) for the severity or frequency of certain behaviors. For example, 7 questions ultimately clustered to generate the Agitation domain (highlighted in Supplementary Table S1). We used a scree plot and Horn’s Parallel Analysis to determine how many components to retain. (Supplementary Fig. S1). Four domains have been identified and the following major symptoms are included in each domain: (1) Agitation: including persistent irritable affect, outbursts, threats of physical harm, and physical violence (TC1, blue rectangle). (2) Affective: including depressed mood, anxiousness, feeling gloomy, weeping, feeling self-critical, feeling guilty and being upset (TC2 green rectangle). (3) Apathy: low level of interest in previous hobbies, loss of interest to engage with others, low motivation to initiate movement, and low emotional responsiveness (TC3, red rectangle). (4) Psychosis: including hallucinations, disinhibition and aberrant motor behavior but not delusions (TC4 orange rectangle), (Supplementary Fig. S1). The domains we found are similar to previous reports [22]. However, in our study, we did not include changes in sleep, appetite, and euphoria [22].

### Sample selection for molecular analyses

To best capture relationships between antemortem BPSD and transcriptional changes, we only considered samples from subjects with BPSD data collected within 2 years of death. Of the initial 192 subjects, we identified 100 subjects who met this criterion and had tissue available for sequencing. We created a scoring system to estimate the BPSD burden for each of these 100 subjects using an additive composite of individual symptom severity and frequency within each domain. For questions with answer choices of no or yes, a score of 0 or 1 was assigned for each question, respectively. For questions with a frequency component, a low or no frequency received a score of 0, a moderate frequency a score of 0.5, and a high frequency a score of 1. The individual question scores were added together to give a final score for each BPSD domain. This yielded four BPSD burden scores for each individual, one for each domain. Within each domain, we used these burden scores to identify patients who were cases (≥70th percentile), controls (≤30th percentile), or neither (31st–69th percentile), and used these groupings for subsequent transcriptome analyses. This design permitted an individual subject to be considered as a case for one domain but as a control for another, maximizing the sample sizes of cases and controls for each domain. In choosing a subset of subjects for our final sample, we made sure that each domain contained similar proportions of cases to controls and of males to females so as to minimize bias due to overrepresentation of any particular group. We ultimately chose 60 subjects for biochemical analyses (Table 1), the process of selecting these 60 subjects is outlined in Supplementary Fig. S2. Though our unbiased grouping of symptoms did not load delusions onto the psychosis domain, the inclusion of delusions in the final score for psychosis did not change the individual classifications of cases and controls used for biochemical analyses.

**Table 1:** RNA-seq sampling information of cases and controls for different BPSD domains in AD subjects.

Affective Domain				
	Control (*n* = 22)	Case (*n* = 21)	Statistical Test	*P*-value (*α* = 0.05)
Female Gender *n*, (%)				
Female	12 (54.55%)	9 (42.86%)	Fisher’s exact test	NS
Age at Death (years)				
M (SD)	81.24 (7.58)	81.07 (8.31)	Welch’s *t* = 0.07 (df = 40.22)	NS
Braak Stage *n*, (%)				
IV	2 (9.09%)	3 (14.29%)	χ²(2) = 1.97	NS
V	8 (36.36%)	11 (52.38%)		
VI	12 (54.55%)	7 (33.33%)		
CERAD Score *n*, (%)				
2	3 (13.64%)	1 (4.76%)	Fisher’s exact test	NS
1	19 (86.36%)	20 (95.24%)		
NIA Raegan Score *n*, (%)				
2	5 (22.73%)	3 (14.29%)	Fisher’s exact test	NS
1	17 (77.27%)	18 (85.71%)		
PMI (Hours)				
M (SD)	5.01 (1.67)	6.51 (5.36)	Welch’s *t* = 1.23 (df = 23.71)	NS

Apathy Domain				
	Control (*n* = 23)	Case (*n* = 24)	Statistical Test	*P*-value (*α* = 0.05)
Female Gender *n*, (%)				
Female	12 (52.17%)	13 (54.17%%)	Fisher’s exact test	NS
Age at Death (years)				
M (SD)	81.90 (8.83)	79.51 (8.13)	Welch’s *t* = 0.96 (df=44.31)	NS
Braak Stage *n*, (%)				
IV	4 (17.39%)	1 (4.17%)	χ²(2) = 6.62	*p* < 0.05
V	7 (30.43%)	16 (66.67%)		
VI	12 (52.17%)	7 (29.17%)		
CERAD Score *n*, (%)				
2	4 (17.39%)	2 (8.33%)	Fisher’s exact test	NS
1	19 (82.61%)	22 (91.67%)		
NIA Raegan Score *n*, (%)				
2	6 (26.09%)	3 (12.50%)	Fisher’s exact test	NS
1	17 (73.91%)	21 (87.50%)		
PMI (Hours)				
M (SD)	7.10 (5.59)	5.94 (3.18)	Welch’s *t* = 0.87 (df = 34.57)	NS

HIDA Domain				
	Control (*n* = 27)	Case (*n* = 22)	Statistical Test	*P*-value (*α* = 0.05)
Female Gender *n*, (%)				
Female	14 (51.85%)	10 (45.45%)	Fisher’s exact test	NS
Age at Death (years)				
M (SD)	81.00 (7.56)	78.71 (7.76)	Welch’s *t* = 1.04 (df = 44.59)	NS
Braak Stage *n*, (%)				
IV	4 (14.81%)	2 (9.09%)	χ²(2) = 0.39	NS
V	12 (44.44%)	10 (45.45%)		
VI	11 (40.74%)	10 (45.45%)		
CERAD Score *n*, (%)				
2	4 (14.81%)	1 (4.54%)	Fisher’s exact test	NS
1	23 (85.19%)	21 (95.45%)		
NIA Raegan Score *n*, (%)				
2	7 (25.93%)	2 (9.09%)	Fisher’s exact test	NS
1	20 (74.07%)	20 (90.91%)		
PMI (Hours)				
M (SD)	6.96 (5.01)	5.33 (2.10)	Welch’s *t* = 1.53 (df = 36.30)	NS

Psychosis Domain				
	Control (*n* = 25)	Case (*n* = 20)	Statistical Test	*P*-value (*α* = 0.05)
Female Gender *n*, (%)				
Female	13 (52%)	10 (50%)	Fisher’s exact test	NS
Age at Death (years)				
M (SD)	82.18 (7.62)	78.91 (7.09)	Welch’s t = 1.49 (df = 41.98)	NS
Braak Stage *n*, (%)				
IV	3 (12%)	1 (5%)	χ²(2) = 0.72	NS
V	12 (48%)	11 (55%)		
VI	10 (40%)	8 (40%)		
CERAD Score *n*, (%)				
2	4 (16%)	0 (0%)	Fisher’s exact test	NS
1	21 (84%)	20 (100%)		
NIA Raegan Score n, (%)				
2	7 (28%)	1 (5%)	Fisher’s exact test	NS (*p* = 0.06)
1	18 (72%)	19 (95%)		
PMI (Hours)				
M (SD)	6.10 (2.67)	6.12 (5.54)	Welch’s *t* = 0.01 (df = 26.01)	NS

The composition of case and control groups within the respective affective, apathy, agitation, and psychosis domains was evaluated for differences in biological sex, age at death, Braak stage, CERAD score, NIA-Reagan score, and postmortem interval. No significant differences in these measures were detected between groups, with the exception of fewer Braak stage IV samples in cases of apathy (*n* = 1) compared to controls (*n* = 4), *p* < 0.05.

### RNA isolation and sequencing

We confirmed four predominant BPSD clusters and then performed bulk RNA-seq analysis on the post-mortem ACC from a subset of 60 individuals with AD with varying burdens of BPSD. The cross-classification of cases for each BPSD domain is summarized in Table 2. Total RNA was isolated using the QIAGEN RNeasy column-based purification kit (Germantown, MD). The quality of RNA was measured using an Agilent Bioanalyzer, which produces an RNA Integrity Number (RIN) between 1 and 10, with 10 being the highest quality samples showing the least degradation. The RINs of the 60 samples ranged between 5.3-10.0 (88% >7.0), and 1 µg of high-quality RNA per sample was used for the total RNA-Seq library preparation. RNA-Seq was conducted at the Northwestern University NUSeq Core Facility. Briefly, the Illumina TruSeq Stranded Total RNA Library Preparation Kit was used to prepare sequencing libraries. The Kit procedure was performed without modifications. This procedure includes rRNA depletion, remaining RNA purification and fragmentation, cDNA synthesis, 3’ end adenylation, Illumina adapter ligation, and library PCR amplification and validation. Illumina HiSeq 4000 Sequencer was used to sequence the libraries with the production of single-end 50 bp reads.

**Table 2:** BPSD Domain Cross-classifications.

Domain/Cross-Classification	Affective	Agitation	Apathy	Psychosis
Affective (*n* = 22)		16 (72.7%)	8 (36.4%)	11 (50.0%)
Agitation (*n* = 23)	16 (69.6%)		12 (52.2%)	15 (65.2%)
Apathy (*n* = 25)	8 (32.0%)	12 (48.0%)		12 (48.0%)
Psychosis (*n* = 21)	11 (52.4%)	15 (71.4%)	12 (57.1%)	

Cases for each BPSD domain (green) are presented with respect to the domains in which the designation of case was also applied (gray).

### RNA-seq differential expression analysis

Raw data was pre-processed with TrimGalore, including an initial quality control (QC). Read depth ranged from 50–90 M. Pseudo-alignment was performed with Kallisto [38] with k-mer 17 due to short read length (50 bp). Genes were pseudoaligned to Genome Reference Consortium Human Build 38. Genes with >80% of samples with total counts <5 were removed. Principal variable component analysis (PVCA) [39] was used to identify likely important covariates by identifying factors that explained a significant proportion of variance. RIN, sex, and RNA-isolation batch were identified as contributing significantly to variation and were included in the final model, while other variables including post-mortem interval, Braak, CERAD, NIA-Reagen scores, and age at death did not. This was also analyzed with Eigen-R2 [40], which largely corroborated the PVCA conclusions. Principal component analysis (PCA) was performed and the first two principal components were visualized in a scatter plot to identify likely outliers. Clustering analysis within WGCNA was also performed and agreed that one sample was a clear outlier and not used in subsequent analyses, leading to 59 total samples analyzed. Final n per group are as follows: Affective domain, *n*_control_ = 23, *n*_case_ = 22; Apathy domain, *n*_control_ = 23, *n*_case_ = 25; Agitation domain, *n*_control_ = 28, *n*_case_ = 23; Psychosis domain, *n*_control_ = 24, *n*_case_ = 21. Because overlapping design allowed for individual samples to be analyzed as a case or control depending on their individual domain score, the sum of all comparisons should not add up to the total 59 included samples. We were underpowered to perform differential expression analysis by sex, and therefore performed 4 independent analyses, one for each domain, using the cases and controls identified by our pre-mortem scoring system. DESeq2 [41] was used to perform differential expression analysis, and a liberal cutoff value of nominal *p* < 0.05 and fold-change >0.2 was used to identify DEGs. Certain DEGs with unusually high variance and fold change were inspected for outliers, and if an isolated datapoint was >3 standard deviations from the mean, the mean was imputed for that point. Visualization of DEG overlap was facilitated with R package VennDiagram.

### Functional enrichment analysis

Functional Enrichment Analysis was performed using gProfiler2 [42], and is described in more detail in supplementary materials.

### Cellular decomposition

Prior to cellular decomposition, WGCNA, and NetDecoder analyses, counts were converted to transcripts per million and underwent covariate correction and variance stabilizing transformation via limma [43]. BRETIGEA [44] was then performed to estimate the relative abundance of 6 different cell types—Astrocytes, Endothelial Cells, Microglia, Neurons, Oligodendrocytes, and Oligodendrocyte Precursor Cells (OPCs)—using 50 different gene markers per cell type. These results were verified with BisqueMarker [45], which did not yield significantly different trends in predicted cell type composition.

### WGCNA

Weighted gene co-expression network analysis (WGCNA) was performed to identify modules of gene co-expression [46, 47]. We included only the top 20% most variable genes by overall expression. Details regarding WGCNA optimization and execution are described more fully in supplementary materials. A potential hub gene was defined similar to previous guidance by WGCNA creators as having a module membership (MM) > 0.8 and gene significance (GS) of >0.2. The notable hub genes in Fig. 3B were identified based on considerations of their high MM, high GS, overall high fold change in expression between cases and controls, and significant presence in the literature as affecting either behaviors in a particular domain or relevance to AD pathogenesis.

### RNA fluorescent barcoding

RNA fluorescent barcoding was used to perform multiplex measurement of 37 agitation domain genes of interest (GoIs), the selection of which was informed by WGCNA and differential expression analysis. A custom CodeSet/ProbeSet (NanoString Technologies, Seattle, WA) was designed to measure GoI transcript counts from the RNA samples that remained available following use for RNAseq (*N* = 47). In addition to GoIs, five reference genes (*IMPDH2, LAMTOR1, MTFR1L, SMIM7, TMEM50B*) were selected based on low covariance between cases and controls in our RNA-seq experiment. Eight negative controls and six positive controls (NanoString Technologies) were measured as a component of QC.

Hybridization of reporter and capture probes to the RNA samples was conducted in accordance with the manufacturer’s protocol (NanoString Technologies, MAN-10056-04). Briefly, 50 ng of total RNA at a concentration of 10 ng/µL was incubated with a Reporter CodeSet-hybridization buffer (NanoString Technologies, item no. 000136) master mix and Capture ProbeSet in a thermocycler at 65 °C for 24 h. Incubation temperature was then reduced to 4 °C until sample processing on the following day. Hybridized samples were brought to a volume of 30 µL with RNAse-free water and loaded into an nCounter SPRINT cartridge (NanoString Technologies, item no. 100078), which was run on an nCounter SPRINT profiler. Transcript counts detected by barcode visualization in the nCounter SPRINT profiler were analyzed using nSolver Analysis Software (v4.0). Differences between group means were evaluated using Welch-Satterthwaite *t*-tests and a threshold of *p* < 0.05 was implemented for determination of statistical significance. All 47 measured samples were included for analysis, as binding density QC indicated sufficient RNA abundance without lane oversaturation, no fields of view were lost during imaging, and assessment of positive control linearity yielded r^2^ = 1.0 for each sample.

### NetDecoder

NetDecoder was performed to compare context-dependent changes in information-flow through case and control networks, as previously described [48]. A fuller description of NetDecoder is provided in supplementary materials. Briefly, we defined DEGs for each domain as source genes, and used iRefIndex v14.0 to build our interaction network. We presented the top 20 positive and top 20 negative genes in terms of flow difference or impact score for visualization across the three intermediary gene types. For visualizing changes in overall domain networks or subnetworks relating to the intermediary genes, we used Cytoscape. To simplify visualization, we filtered out edges where there was very little difference in flow, thus highlighting the results with the largest effects on the networks.

## Results

### BPSD segregate into four domains

**Fig. 1:**
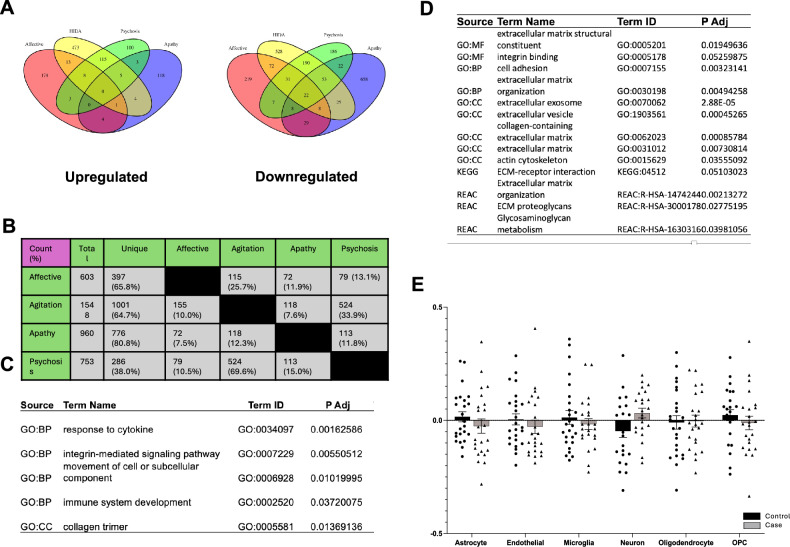
Each BPSD domain is associated with unique differentially expressed genes. **A** Venn diagrams detailing overlap between DEGs in each domain. DEGs were defined by a fold change >0.20 and a nominal *p* < 0.05. **B** Table depicting numbers of DEGs that are either shared between two domains or uniquely expressed. Percentages represent how many DEGs are in each category compared to the total number of DEGs. **C** Functional gene enrichment analysis (FGEA) of the 22 DEGs that were commonly downregulated in all four BPSD domains. The most salient pathways were chosen for this table. **D** For the agitation domain, FGEA of all DEGs associated with this domain. The most salient pathways were chosen for this table. **E** Relative cell abundance estimated with BRETIGIA. After correcting for multiple comparisons, there were no differences in cell type between cases and controls for the Agitation Domain (*p* < 0.05).

Though BPSD are heterogeneous, previous reports have indicated that common symptoms often co-occur at high rates and can be grouped into domains [21, 22]. However, while groupings for BPSD are generally consistent across studies, there are slight variations that could be related to the specific cohort studied (i.e., community, nursing home, assisted living facility, etc.) or stochasticity. We performed clustering analysis of BPSD based on data from a clinical cohort where the frequency and severity of individual BPSD within 2 years from the patients’ deaths was recorded. We determined grouping of BPSD into the following four domains: affective, psychosis, agitation, and apathy (Fig. 1). The proportions of variance explained by each factor were comparatively similar. Delusions tended not to be explained by any single loading factor, so these were not included in the psychosis domain, although these are often grouped together clinically and in prior factor analyses [22].

### Unique molecular signatures associate with each BPSD domain

After demonstrating BPSD could be split into four domains, we created a scoring system to estimate BPSD burden using a composite of individual symptom severity and frequency within each domain and grouped individual as cases or controls for each domain. Though BPSD are likely to result from dysfunction of multiple brain regions, the ACC has repeatedly been implicated as being involved in all four BPSD domains [49–51]. Therefore, we performed bulk RNA-seq on ACC tissue from a subset of individuals from our cohort that maximized the numbers of individuals who could be considered a case or control (Supplementary Table 1).

Using a liberal cut-off for significance (nominal *p* < 0.05 and fold change >0.20), we identified hundreds of DEGs for each BPSD domain with patterns of downregulated DEGs for all four domains. Specifically, we found 207 upregulated and 396 downregulated (66% of total) DEGs for the affective domain; 135 upregulated and 788 downregulated (86%) DEGs for the apathy domain; 619 upregulated and 931 downregulated (60%) DEGs for the agitation domain; and 227 upregulated and 519 (70%) downregulated DEGs for the psychosis domain (Fig. 1A).

Importantly, despite an experimental design using the same subjects as cases and controls on a domain-by-domain basis, most of the DEGs were transcriptionally unique for each domain (Fig. 1B): 65.8% unique for the affective domain, 80.8% unique for the apathy domain, and 64.7% unique for the agitation domain. The exception was the psychosis domain, where only 38.0% of DEGs were unique due to high overlap with DEGs from the agitation domain.

To further explore the correlations between the differential expression of each behavioral domain, we performed rank-rank hypergeometric overlap (RRHO) analyses [52]. Nearly all the pairs of behavioral domains showed concordance, especially for genes mutually downregulated in each behavioral case compared to controls. In general, the degree of concordance scaled with the degree of overlap of DEGs. For instance, the highest concordance was found for the agitation and psychosis domains, which also showed the greatest degree of overlapping DEGs. In contrast, the apathy and agitation domains showed moderate concordance for mutually downregulated genes but little concordance for mutually upregulated genes, mirroring the low rate of overlap in DEGs between these behavioral domains. In fact, some genes downregulated in the apathy domain were upregulated in the agitation domains (Supplementary Figs. S3, S4).

Transcriptional signatures can be used to estimate the relative cell abundance of the origin tissue. Therefore, we used BRETIGEA to estimate the cell abundance of 6 major cell types—astrocytes, endothelial cells, microglia, neurons, oligodendrocytes, and OPCs. Across all four domains, only a decrease in microglia was detected for cases compared to controls in the apathy domain, while all other cell type compositions were comparable (*p* < 0.05; Supplementary Fig. S3). The results were similar when a different deconvolution algorithm, BisqueMarker, was used (data not shown).

Interestingly, there were only 22 DEGs that were shared by all four BPSD domains (<3.6% of total DEGs in any domain) and all were downregulated. Functional enrichment analysis yielded pathways related to response to cytokines, integrin-mediated signaling, and collagen trimers (Fig. 1C), with *TIMP1* being a notable DEG (M_Fold Change, agitation_. = 0.644, *p* = 3.0 × 10^−5^; M_FC, affective_ = 0.748, *p* = 0.012; M_FC, apathy_. = 0.644, *p* = 1.1 × 10^−5^; M_FC, psychosis_. = 0.642, *p* = 1.1 × 10^−5^).

Functional enrichment analysis of the DEGs were performed for each BPSD domain. Here, we focus on the results of the agitation domain, as this domain is often the most challenging for families and clinicians due to the high frequency of safety issues including aggression, increased risk-taking, and impulsivity (see Supplementary Materials for results and discussion of the other 3 BPSD domains). We found an enrichment for the extracellular matrix (ECM) including actin, collagen, glycosaminoglycans, extracellular vesicles, and cellular adhesion (Fig. 1D). Transcriptomic changes detected in agitation cases did not coincide with differences in the abundance of any individual cell type (Fig. 1E).

**Fig. 2:**
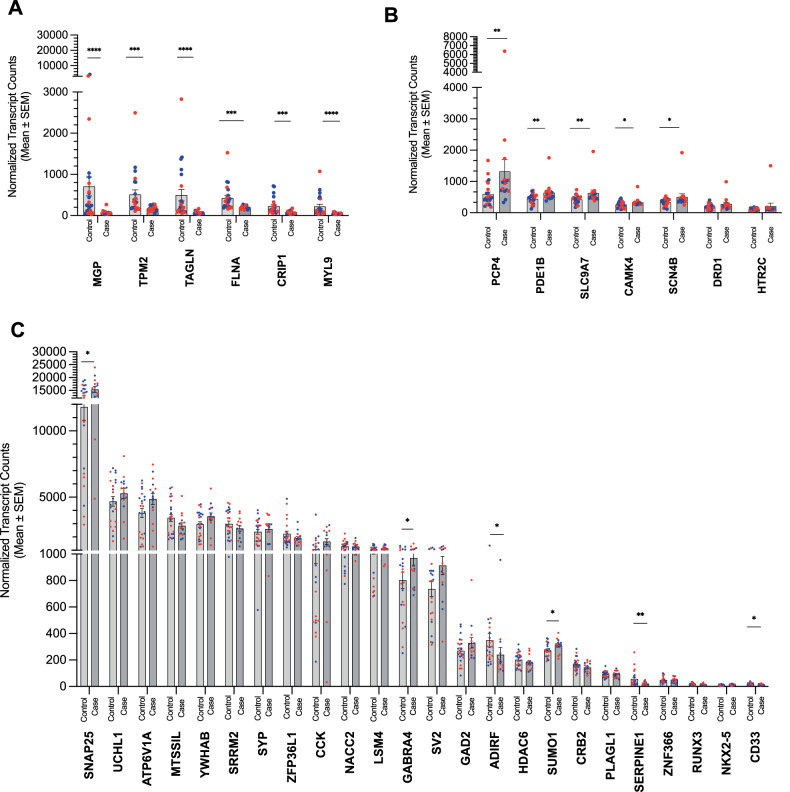
Validation of key DEGs for the agitation domain. The differential expression of (**A**) Six DEGs that are also potential hub genes in the ECM (Darkturquoise) module and (**B**) Five DEGs that are potential hub genes in the post-synapse (Darkgrey) module were confirmed with RNA fluorescent barcoding after expression was normalized to five housekeeping genes. **C** Non-WGCNA genes and modules with a single confirmed potential hub gene (i.e., *ADIRF* from the nucleosome (Lightyellow) module and *CD33* from the transcription factor (Magenta) module) are represented. Blue circle = male, red circle = female; ****p < 0.0001, ***p < 0.001, **p < 0 .01, *p < 0.05.

To confirm some of the results for our agitation domain, we quantitated transcripts using Nanostring. Though our statistical power was more limited than our initial RNAseq, we were able to confirm 17/37 genes to be significantly different between cases and controls for the agitation domain, including *TIMP1, TAGLN*, and *FLNA* (Fig. 2).

### Transcriptional modules are unique and shared among BPSD domains

**Fig. 3:**
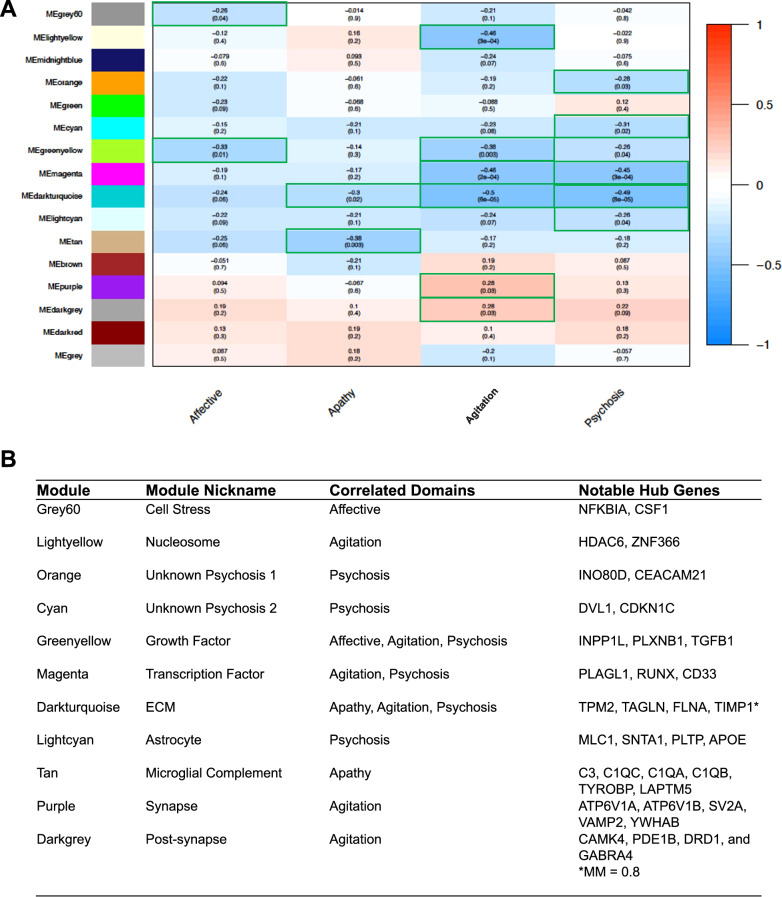
Co-expression modules associated with each BPSD domain. **A** Heatmap of co-expression modules using WGCNA. Color of each module is arbitrarily chosen, except for the gray module, which represents genes that did not correlate with the expression of other genes. The top number in each square represents the Pearson’s r while the *p*-value is in the parenthesis underneath. Red color squares have a positive correlation with BPSD status while blue squares have a negative correlation, and the intensity of shading scales with increasing Pearson’s r. **B** Table of module names, nicknames, and notable hub genes. All notable hub genes had module membership (MM) > 0.80, except where specified. Gene nicknames were chosen based on the predominant pathways implicated in the functional enrichment analyses.

Genes that are transcribed similarly often regulate similar biological processes, and it can be informative to group genes into co-expression modules that suggest co-regulation. In addition, these co-expression analyses can yield genes that are highly connected to the rest of the network, termed hub genes, that may be central to the transcriptional network and therefore of high interest mechanistically. We utilized WGCNA across all transcriptomes and associated modules linked with the case/control condition for each BPSD domain. As expected, some modules were shared across certain domains, especially for psychosis and agitation, and other modules were uniquely significant for a single domain (Fig. 3A, Supplementary Fig. S6). In particular, an 88-gene module for growth factor and cell adhesion (greenyellow) was shared across the affective, agitation, and psychosis domains; a 28-gene module for ECM and actin cytoskeleton (darkturquoise) was shared across apathy, agitation, and psychosis; and a 98-gene module for transcription factor activity (magenta) was shared across agitation and psychosis; no module was associated with all four domains, again suggesting the separability of these traits on a transcriptional level. All shared modules suggested a reduction in transcription in cases, consistent with differential expression trends. Focusing on the agitation domain, three modules were uniquely associated: modules for nucleosome assembly (lightyellow; 35 genes), ATPase and synaptic signaling (purple; 916 genes), and post-synaptic signaling and response to monoamines (darkgrey; 63-gene). The post-synaptic and monoamine module was enriched for serotoninergic signaling, dopaminergic signaling, and GABAergic signaling. Interestingly, the synaptic signaling modules (purple and darkgrey) were amongst the only significantly associated modules that demonstrated increased transcription for cases.

Hub genes in WGCNA that are associated with a given domain are defined by the high connectivity within their module as well as significant influence on transcription with the BPSD domain (Fig. 3B). The ECM module, associated strongly with agitation and psychosis and more weakly with apathy, yielded 10 potential hub genes, including *TAGLN* (M_FC,agitation_ = 0.362, *p* = 1.3 × 10^−5^) and *FLNA* (M_FC, agitation_ = 0.646, *p* = 5.9 × 10^−6^). *TIMP1* was also in this domain but had a module membership value slightly below the cut-off for potential hub genes. The synaptic signaling and monoamine module (darkgrey), a unique module with increased transcription for aggression domain cases, had 39 hubs genes including *CAMK4* (M_FC, agitation_ = 1.29, *p* = 0.022), *PDE1B* (M_FC, agitation_ = 1.39, *p* = 0.007), *DRD1* (M_FC, agitation_ = 1.38, *p* = 0.006), and *GABRA4* (M_FC, agitation_ = 1.26, *p* = 0.019).

### Potential drivers of transcriptional network information flow with the agitation domain

BPSD likely arise from complex changes in multiple biological networks, and the dynamic interactions of genes and proteins within a network may be key to understanding the difference between the BPSD cases and controls in AD. While enrichment analyses suggest important pathways based on the overrepresentation of genes compared to chance, they cannot inform how biological information may change in a context-dependent manner—such as a disease vs non-disease state. Therefore, we used NetDecoder to compare the “information-flow” through each BPSD domain’s cases and controls’ networks, which utilizes a process-guided flow algorithm to identify the weights of information flow from source genes, DEGs, to target genes and transcriptional regulators [48]. For context, when this algorithm was applied to an older breast cancer dataset, they were able to identify three later-confirmed, prognostic markers as important drivers of information flow in the cancer network that were not originally implicated in the original study that lacked a context-dependent approach. Important genes in NetDecoder are labeled as high-impact genes, network routers, or key targets, and by a function of the algorithm, all are genes that were not identified as DEGs but likely affect overall information-flow through the biological system via regulation of downstream transcription.

**Fig. 4:**
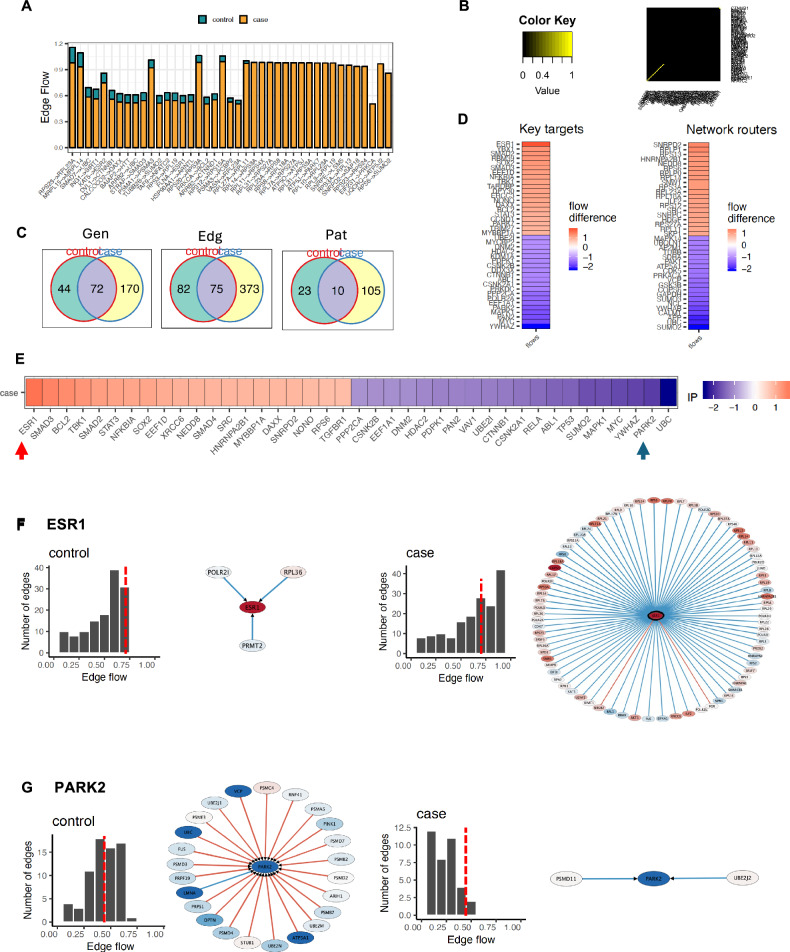
Divergent information flow between transcriptional networks in the agitation domain. **A** Total edge flow profiles in control versus case subnetworks for the agitation domain. Overall, edges display decreased flow in cases versus controls. **B** Jaccard index evaluating similarity between case and control subnetworks. **C** Venn diagrams depicting overlap (blue) in genes, edges, and paths between cases (yellow) and controls (green). **D** Heatmaps showing top 20 network routers and key targets with the most increased (red) and most decreased (blue) flow difference. Network routers are intermediary genes with the highest difference in flow when comparing the case and control subnetworks. Key targets are transcriptional regulators that act as ‘sinks’ in this analysis and have the highest difference in flow to them when comparing the case and control subnetwork. **E** Top 20 Impact Genes with the most increased (red) and most decreased (blue) flow differences between case and control subnetwork. Impact scores to determine top impact genes are based on total flow difference at each node between cases and controls, proportion of newly established interactions in the case subnetwork, and number of edges where the expression correlation changes directionality from control to case. Arrows point to notable genes depicted in the rest of figure (below) and main text. **F** Differences in information flow between control and case subnetworks at *ESR1* and (**G**) *PARK2*. Nodes are colored according to the node flow differences across case and control subnetworks. Edge thickness, detailed on the histogram’s *x*-axis, represents total amount of edge flow from 0 to 1. The direction of flow is determined by overall structure of information flow from source to sinks. A red edge depicts a positive gene expression correlation between a pair of protein-protein interactions, while blue edges represent a negative correlation. Histograms depict the number of edges binned by total flow. Genes with flow amounts to the right of the dashed, red line are depicted in the corresponding graphic to the right of each histogram.

Focusing on the agitation domain, NetDecoder revealed divergent information flow between cases and controls, and the top 40 network routers, key targets, and high-impact genes are shown (Fig. 4A–E; Supplementary Fig. S4). Though a number of high-impact genes are of interest, two are particularly notable (Fig. 4E, Arrows indicated**)**. The ER-beta, encoded by *ESR1*, was identified as the top key target and high-impact gene mediating positive information flow (Fig. 4F). While little remains known about *ESR1* function in agitation/aggression in the frontal cortex, *ESR1*^+^ cells within the hypothalamus and other limbic regions have robust evidence linking them to control of aggressive behaviors [53–58]. The other notable key target is Parkin, the ubiquitin ligase encoded by *PARK2* (Fig. 4G) and genetically associated with familial Parkinson’s disease. *PARK2* has significant cross-talk with tau, regulates mitophagy in AD, and interestingly has been linked to impulsive behaviors in Parkinson’s disease [59–61].

## Discussion

The data presented here represent the first exploration of affective, apathy, and agitation symptoms on the transcriptome-wide level and establish unique patterns of mRNA expression in one of the brain regions most consistently implicated with BPSD, the ACC [49–51]. In addition, we add to the growing knowledge base about transcriptional changes in AD with psychosis [25] (discussed along with the affective and apathy domain in supplementary materials). We confirmed that commonly co-occurring BPSD cluster into domains in our cohort and that these domains are typified by unique transcriptional signatures in the ACC, even when individual samples are used in an overlapping design. Using co-expression analyses, we observed individual BPSD domains being associated with shared and unique transcriptional modules with potential hub genes that may serve as targets for future discovery of discrete molecular mechanisms and novel pharmacology. Finally, we identified key drivers of information flow through biological networks associated with BPSD domains, highlighted by *ESR1* and *PARK2* being potential mediators of the agitation domain in AD.

Though BPSD may be thought of as manifestations of late-life primary psychiatric disorders (PPD), there is already some evidence suggesting that BPSD mechanisms diverge molecularly. There have been a handful of genetic studies suggesting overlapping polygenic risk for PPD and neurodegenerative disease [62, 63], and while good epidemiological evidence suggests PPD are risk factors for resultant neurodegenerative dementias [64, 65], the few studies comparing PPD and BPSD on a genetic level have yielded surprising results. For instance, while psychosis—focusing on the ‘positive’ symptoms of hallucinations and delusions—is a prominent symptom in schizophrenia, bipolar disorder, and AD with psychosis, a recent GWAS found negative correlations between schizophrenia and AD with psychosis and bipolar disorder and AD with psychosis, suggesting not just a lack of association between these PPD and BPSD, but a reduced risk of psychosis in AD with increased polygenic risk for schizophrenia or bipolar disorder [66, 67]. This is a stark contrast to the extensive genetic overlap between schizophrenia and bipolar disorder risk [68], suggesting that BPSD may be mechanistically distinct from PPD.

Treatment of BPSD has also yielded surprising differences from PPD. For instance, the HTA-SADD trial found no benefit of two common serotonergic antidepressants for depression in AD [69], and a Cochrane Database meta-analysis supported this lack of efficacy [70]. Similarly, while selective serotonin reuptake inhibitors (SSRIs) are not used to treat hallucinations and delusions in PPD for psychotic disorders, and may even induce psychosis in bipolar disorder through increasing the risk of mania [71], a common SSRI citalopram seems to have some benefit for reducing these psychotic symptoms in AD, though this was discovered on secondary analysis and requires follow-up [72]. Similar to genetic differences between BPSD and PPD, it seems likely that pharmaceutical approaches need to differ substantially in treating the two disorders, which necessitates the further investigation of BPSD as its own entity, distinct from PPD.

Rigorous characterizations of BPSD ante-mortem with complementary post-mortem tissues for molecular analyses are remarkably scarce, so we sought to optimize our analytical power for the samples we were able to obtain. In addition, given the high prevalence of BPSD in AD (>95%), finding enough controls without any BPSD would be very challenging. This required us to adopt an experimental design where each individual’s sample could be considered to be a case (30% highest score of the domain) or control (30% lowest score of the domain) for a specific BPSD domain, meaning that some transcriptome could be potentially analyzed multiple times depending on the comparison. Therefore, it was encouraging to see such a large number of DEGs that are unique to each domain despite the overlapping design, which may suggest that each BPSD domain has distinct biological etiologies despite the common neuropathological drivers – in this case, AD. These findings may be comparable to other investigations showing distinct pathways associated with cognitive decline in AD at the transcriptomic, proteomic, and methylation levels in brain tissue without overlapping correlation with AD pathology [73–77], highlighting the heterogeneity of downstream molecular processes from what is putatively considered the upstream etiological agents, namely Aβ and tau. Though this design has the advantage of increasing statistical power in exploring a wide array of symptoms, it presumes separability that precludes the identification of individuals with overlapping psychiatric domains that may have unique molecular mechanisms distinct from if these symptoms are presented separately. Future investigations can help clarify this.

Given this overlapping design, it was surprising that so few genes were shared amongst all the BPSD domains. It is notable that response to cytokines was implicated as a functionally enriched pathway, as neuroinflammation is often considered an integral driver of neurodegeneration and subsequent synaptic dysfunction [78]. *TIMP1* was among the 22 shared DEGs, all of which were downregulated. A major inhibitor to a number of metalloproteases such as MMP3 and MMP9, *TIMP1* has been implicated in multiple forms of neurodegeneration and neuroinflammation [79, 80], with the hypothesis that early upregulation of *TIMP1* maintains balance in neurodegenerative states while late downregulation may suggest an inability to achieve homeostasis [81]. It is therefore speculative but possible that reduced expression of *TIMP1* leads to loss of homeostasis, which could lead to heightened stochasticity in downstream processes and divergent BPSD. While it is also interesting that *TIMP1* has been suggested as a biomarker in biofluids in both Parkinson’s disease and AD [82–84], further exploration of *TIMP1* could be especially fruitful in understanding the early drivers of BPSD.

Though our analyses discovered multiple interesting targets that were unique to each BPSD domain (see supplementary materials for in-depth results and discussion), we focused on the results of the agitation domain. The enrichment analysis of the DEGs for this domain were highly suggestive of changes in the ECM or matrisome, and a shared transcriptional module associated with agitation, apathy, and psychosis similarly was enriched for the ECM. A recent and extensive proteome-wide study in AD found that a module of co-expressed proteins enriched for the matrisome was highly correlated with global AD pathology and ApoE status but shockingly independent of cognition [85]. The possibility exists that changes in the ECM are less associated with cognition but are better tied to BPSD, especially agitation. Interestingly, a common functional SNP in MMP9, which is inhibited by *TIMP1*, was associated with inhibition of aggression and irritability in one study [86]. Currently, much more evidence would be needed to link changes in the ECM with agitation in AD, but this finding further highlights the necessity of considering BPSD in studies of neurodegeneration in addition to cognition, as there may be diverging molecular mechanism related to both sets of symptoms.

The module for post-synaptic signaling and monoamines was upregulated in agitation cases versus controls and was enriched for genes that are common treatment targets for agitation, such as those related to dopamine (antipsychotics), serotonin (antipsychotics and antidepressants), and GABA-A receptors (benzodiazepines) [20]. As monoaminergic treatments are so important in PPD but have limited to no success for the affective domain, mainly depression [69, 70], it was interesting to note that none of the serotonergic, dopaminergic, adrenergic, or muscarinic receptors were DEGs for the affective domain. In contrast, *DRD1* and *5HTR2C* were DEGs for the psychosis and agitation domains while the psychosis domain also demonstrated *DRD2* and *DRD4* as DEGs. While far from conclusive, this again dovetails with the treatment failures for SSRIs for affective behavior in BPSD and further supports the hypothesis that PPD have fewer mechanistic similarities to BPSD.

Additionally, the finding that DRD1, a GPCR coupled to Gs/α, and downstream effectors PDE1B and CAMK4 are associated with this module and the agitation domain is in line with some genetic reports that *DRD1* SNPs are associated with greater impulsivity and aggression [87, 88], including in AD [89, 90] and Parkinson’s disease [91]. The role of striatal DRD1 in aggression has been demonstrated before [92], but how DRD1 and some of its downstream signaling molecules affect agitation/aggression in the ACC is less clear. Similar to DRD1, increased GABA-A signaling in the prefrontal cortex, including the ACC, has been linked with increased aggressive and impulsive behaviors [93] and may interact with CAMK4 expression in certain situations [94]. It was notable that those with significant agitation domain behaviors trended towards having more neurons in the ACC than controls, though this analysis precluded investigation of the neuronal subtypes. It is possible that relative increases in GABAergic neurons in cases or decreases in glutamatergic neurons in controls could lead to these differences in agitation domain behavior.

Transcriptome datasets are inherently noisy, but the pattern of differential gene expression can be combined with a priori knowledge of protein-protein interactions to uncover hidden drivers of disease that affect the transcriptional regulation network. Using an analytical framework to analyze transcription in this context-dependent way, we uncovered two high-interest gene targets related to the agitation domain, *ESR1* and *PARK2*. For neurons in the ventromedial hypothalamus [56], posterior and medial amygdala [58], and bed nucleus of the stria terminalis [55], *ESR1*^*+*^ expression differentiates these cells as ones that regulate aggression from the behavioral function of *ESR1*^*-*^ cells. Despite the importance of *ESR1* as a marker of aggression-regulating neurons, the actual function of *ESR1* in the cell and resultant aggressive behavior is less clear, though knockout of *ESR1* in mice leads to reduced aggression [95] and *ESR1* polymorphisms have been linked with aggression in humans [96] and songbirds [97]. Understanding *ESR1*’s role in the ACC in terms of agitation, aggression, and impulsivity may be a particularly fruitful avenue for mechanistic understanding and future drug development.

The main novelty of our study is that it is the first, to our knowledge, to study the affective, apathy, and agitation domains on the transcriptome-wide level, which is comparable to the many studies of cognition in AD [74, 75], dating back at least as early as 2008 [77], and the recent study of AD with psychosis [25]. Despite this, there are important limitations worth noting. First, our bulk tissue approach precludes a deeper exploration of the role of different cell types in BPSD. Similarly, we present only one brain region’s worth of data, and it is likely that interactions with other regions, such as the posterior cingulate cortex, orbitofrontal cortex, hippocampus, and monoaminergic nuclei, are necessary to fully understand each BPSD domain’s pathogenesis. While our clinical characterizations were robust in capturing the intensity and frequency of many behaviors, results may not be as generalizable to other questionnaires like the Neuropsychiatric Inventory-Questionnaire, which asks about single domain intensity without querying specific behaviors or frequency. While the strength of our approach in assaying BPSD was through structured interviews conducted by well-trained psychiatric providers, we acknowledge that this instrument was not tested for validity against other BPSD instruments nor gold-standard psychiatric assessments; though for certain behaviors (i.e., agitation), these types of gold-standard diagnoses do not exist. Similarly, we cannot exclude potential bias introduced through narrowing our group of subjects for transcriptomic analyses due to constraints on available tissue and timely BPSD assessments. We were also unable to associate our findings with medication data before the patient’s death, which will be an important covariate to include in future studies. Additionally, transcriptomic differences do not always translate into protein differences [85], so future proteomic studies would help solidify the significance of our results. Finally, while our method of unbiased clustering recapitulated similar clustering patterns to the literature (e.g., including disinhibition in the psychosis domain) [22, 98, 99], there are notable differences across cohorts and across studies. For example, some articles included delusion in the psychosis domain [100, 101]. We excluded delusions from this domain in our study because they tended not to be explained by any single loading factor. Given that the exclusion of delusions did not alter the designation of cases and controls, it seems unlikely that any significant enrichments or differential gene expression underlying psychosis in our cohort would have escaped our results as a consequence of our unbiased, data-driven approach. It may, however, be imprudent to use the present results to investigate delusion-specific mechanisms of psychosis in AD. However, we included disinhibition in this domain as others did [98, 99]. While we believe these transcriptional changes are central to perturbed biological mechanisms for each behavioral domain, the individual behaviors included in each cluster may not be directly generalizable to other clustering studies. Even with these limitations in mind, we hope our work will lead to future molecular investigations into BPSD so that advanced therapeutics can be designed and translated to the clinic for many of our society’s most vulnerable and affected patients. We believe it will be particularly important to study those with PPD and no AD neuropathologic change compared to those with AD-related BPSD to further characterize the molecular divergence in these pathologies.

### Supplementary information

Revision_FINAL_Supplementary Materials_BPSD and AD_RNAseq

Supplementary Table S1 Questions and evaluations for behavior and psychological symptoms in AD patients with dementia.

## Data Availability

RNA-seq data used in the analysis and conclusions made in this paper will be submitted to the AD Knowledge Portal and available upon request.
